# Photodegradation of Unsymmetrical Dimethylhydrazine by TiO_2_ Nanorod Arrays Decorated with CdS Nanoparticles Under Visible Light

**DOI:** 10.1186/s11671-016-1718-9

**Published:** 2016-11-10

**Authors:** Xin Gao, Xiangxuan Liu, Xuanjun Wang, Zuoming Zhu, Zheng Xie, Jun Li

**Affiliations:** 1 High-Tech Institute of Xi’an, Shaanxi, 710025 China; 2 High-Tech Institute of Beijing, Beijing, 100085 China

**Keywords:** TiO_2_ nanorod arrays, CdS nanoparticle, Photocatalysis, Visible light, Unsymmetrical dimethylhydrazine

## Abstract

Photocatalysis technology could utilize solar energy to degrade many toxic pollutants and provides possibility to deal with unsymmetrical dimethylhydrazine (UDMH) wastewater with less energy consumption. In this study, well-aligned TiO_2_ nanorod arrays (TiO_2_ NRAs) were grown directly on transparent conductive glass (FTO) via a hydrothermal method, and TiO_2_ NRAs/CdS heterostructure films were prepared by decorating TiO_2_ NRAs with CdS nanoparticles through successive ion layer adsorption and reaction (SILAR). Under visible light, the TiO_2_ NRAs/CdS heterostructure displays enhanced photodegrading capacity compared with the bare TiO_2_ NRAs, and the highest photodegradation rate, 27.5% higher than that of the bare TiO_2_ NRAs, was achieved by the sample with 15 SILAR cycles. Additionally, the solution pH had some influence on the degradation process, which shows that the best degradation rate can be achieved in the neutral solution (pH is ca. 7.2), and the photodegradation process can be better in alkaline solution than in the acid solution. Moreover, the visible photocatalytic stability of the TiO_2_ NRAs/CdS sample was investigated. Finally, the underlying photocatalytic mechanism was discussed according to the photoelectrochemical and photoluminescence results.

**Electronic supplementary material:**

The online version of this article (doi:10.1186/s11671-016-1718-9) contains supplementary material, which is available to authorized users.

## Background

Unsymmetrical dimethylhydrazine (UDMH) is primarily used as a high-energy propellant [1]. However, as an eco-toxicant, UDMH greatly endangers human health once occurred in water under natural condition. Traditional water treatment methods, such as chlorination, ozone oxidation, and catalytic oxidation with oxygen and hydrogen peroxide in the presence of Cu, Fe, and Co salts supported on zeolites as catalysts, achieve satisfactory degrading results, but the high energy consumption (continuous need for an oxidizing agent such as hydrogen peroxide and ozone) and complexity of the recycle of the catalyst make these methods inefficient for applications. Recently, cavitational decontamination of UDMH wastewater seems attractive. Even though it is an oxidant-free method, much energy is still needed to form gas bubbles in the liquid and then explosively develop, grow large, and, at last, collapse [1]. Therefore, it is urgent to develop an energy-efficient method to remove UDMH from water. It is reported that photocatalyst titanium oxide (TiO_2_) is non-selective during degradation of organic compounds [2]. A lot of researches have been made to degrade various organic contaminants and got excellent degrading results [3–9]. In this respect, using TiO_2_ as a photocatalyst to degrade UDMH may be an energy-saving manner.

Furthermore, TiO_2_ is abundant, low cost, nontoxic, and highly resistant to photocorrosion [10]. Once inspired, the generated electron and hole pairs (*e* ^−^/*h* ^+^) migrate to the surface of TiO_2_ for a direct oxidation of the polluting species or undergo redox reactions [11] at the surface of the semiconductor to form extremely reactive oxygen species (·OH, O_2_·, H_2_O_2_, O_3_, etc.) and then degrade the pollutions [12]. In particular, regular one-dimensional TiO_2_, with efficient and tunable optical absorption as well as low reflectivity [13], exhibits good performance due to the unique nanostructure, which facilitates the effective separation of the photoexcited carriers.

However, the band gap (3.0~3.2 eV) of TiO_2_ is too wide to efficiently absorb visible light, which is the main component of the solar spectrum (ca. 43%) [14]. One of the promising strategies to overcome this drawback is to couple TiO_2_ with other narrow band gap semiconductors capable of harvesting the photons in the visible light region [15]. CdS with a band gap of ca. 2.2–2.4 eV [16] has been one of the most intensively studied narrow band gap semiconductors to improve the visible light absorption of TiO_2_. Moreover, the good match of band positions between TiO_2_ and CdS ensures efficient separation of charge carriers [17, 18].

So far, few researches are reported in dealing with UDMH wastewater by semiconductor photocatalysis. Most of the reported researches are conducted under UV irradiation and use powder catalysts. Here, we prepared visible light-induced TiO_2_ nanorod arrays (TiO_2_ NRAs) decorated with CdS thin films and applied the obtained photocatalyst for the degradation of UDMH under visible light irradiation. To the best of our knowledge, research on one-dimensional TiO_2_ NRAs decorated with CdS to degrade UDMH under visible light irradiation has not been reported. Compared with the bare TiO_2_ NRAs, TiO_2_ NRAs/CdS exhibited dramatically enhanced photocatalytic capacity. By adjusting the amount of CdS deposited on the TiO_2_ NRAs, the degrading rate can be improved significantly. The effect of pH of wastewater on the degrading rate was investigated. Finally, photoelectrochemical performance and photoluminescence (PL) spectra were measured to clarify the photocatalytic mechanism.

## Methods

### Synthesis of TiO_2_ Nanorod Arrays Decorated with CdS

Vertically aligned TiO_2_ NRAs were prepared on transparent fluorine-doped tin oxide (FTO) glass substrates (14 Ω/sq) using the hydrothermal method based on our published procedure [19]. Deionized water (DI, 10 mL) was mixed with hydrochloric acid (10 mL, 36.8 wt%) and stirred for 5 min before tetrabutyl titanate (0.4 mL, 98%) was added. When the solution was stirred to clear clarification, the mixture solution was transferred to a Teflon-lined stainless steel autoclave. Clean FTO substrates (area 4.5 cm^2^) were immersed with the conducting side face down. The autoclave was put in an oven at a temperature of 150 °C and taken out from the oven after 5 h. After the autoclave was cooled to room temperature, the FTO substrate was rinsed with DI water and dried naturally at room temperature.

CdS nanoparticles were deposited on TiO_2_ nanorod arrays through a successive ion layer adsorption and reaction (SILAR) method according to the experimental procedure reported by Xie et al. [20] with a slight modification. Briefly, the TiO_2_ NRAs substrate was dipped in a 0.01 M Cd(NO_3_)_2_ aqueous solution for 30 s, rinsing it with DI water for 30 s, and then immersed into a 0.01 M Na_2_S aqueous solution for another 30 s, and rinsing it again with DI water for 30 s. The SILAR process was repeated to obtain TiO_2_ NRAs sensitized with different amounts of CdS nanoparticles, which were designated as TiO_2_ NRAs/CdS (*n* cycles).

### Characterization

The surface morphology was obtained with a scanning electron microscopy (SEM, VEDAIIXMUINCN) equipped with an energy-dispersive X-ray spectroscopy (EDS) system. Waster 5510 transmission electron microscopy (TEM) was used to further characterize the film microstructure. X-ray diffraction (XRD, PANalytical) with Cu-Kα (*λ* = 0.15401 nm) was operated at 40 kV and 40 mA in a 2*θ* range of 20°–80° at a scanning speed of 5° min^−1^. Raman spectra were recorded using an inVia Reflex Raman spectrometer under Ar^+^ (532 nm) laser excitation at room temperature. The optical properties were probed by a UV–vis spectrophotometer (UV1800, Shimadzu) with FTO substrate as a blank. X-ray photoelectron spectroscopy (XPS) was obtained using ESCALAB 250Xi (The binding energy of the XPS spectra was calibrated with reference to the C 1s peak at 284.8 eV.)

Photoelectrochemical measurements were performed in a 250-mL quartz cell using a three-electrode configuration, including the prepared sample as a working electrode, a Pt foil as a counter electrode, a saturated Ag/AgCl as a reference electrode, and 0.1 M Na_2_S as an electrolyte. The working electrode was illuminated within an area of about 1.5 cm^2^ at zero bias voltage versus the Ag/AgCl electrode under solar-simulated (AM 1.5 G filtered, 100 mW cm^−2^, CEL-HXF300) light sources with a UV cutoff filter (providing visible light with *λ* ≥ 420 nm). The photoluminescence (PL) spectra for solid samples were recorded on a Fluoromax-4 spectrophotometer with an excitation wavelength at 350 nm.

### Photocatalytic Degradation of UDMH

The photodegradation of UDMH aqueous solution was carried out in an open reactor under visible light irradiation. The TiO_2_ NRAs/CdS films (area about 6 cm^2^) were immersed in UDMH aqueous solution (15 mL) with an initial concentration of 20 mg L^−1^. Then, dark (adsorption) experiments were carried out for 30 min to reach the adsorption equilibrium of UDMH with the TiO_2_ NRAs/CdS film. The film-coated side of the substrate was exposed to the light source, and the light source was a 300-W xenon lamp with visible light illumination of 60 mW cm^−2^; an ultraviolet cutoff filter was used to exclude UV light with a wavelength below 420 nm. Traces of UDMH can react with amino ferrocyanide sodium to form a red complex in a weakly acidic aqueous solution, and the color depth of the red complex is proportional to the content of UDMH. So, the concentration of UDMH left in the aqueous system can be measured by a spectrophotometer at 500 nm which is the characteristic absorption wavelength of the red complex. The procedure is as follows: (1) UDMH aqueous solution (0.5 mL) was added to a test tube with a volume of 50 mL and then diluted to 25 mL by DI water. (2) Buffer solution (1 mL) was added to adjust the above solution to a weakly acidic aqueous solution. The buffer solution was made of citric acid and disodium hydrogen phosphate with a pH of about 4.8. (3) Amino ferrocyanide sodium (1 mL, 1.5 g L^−1^) was added to the test tube, and then, the test tube was placed in 30 °C water bath for 1 h. (4) The final red complex solution was measured by a spectrophotometer at 500 nm. The relative concentration of UDMH in the solution was derived by comparing its absorption intensity with the standard curve line.

## Results and Discussion

**Fig. 1:**
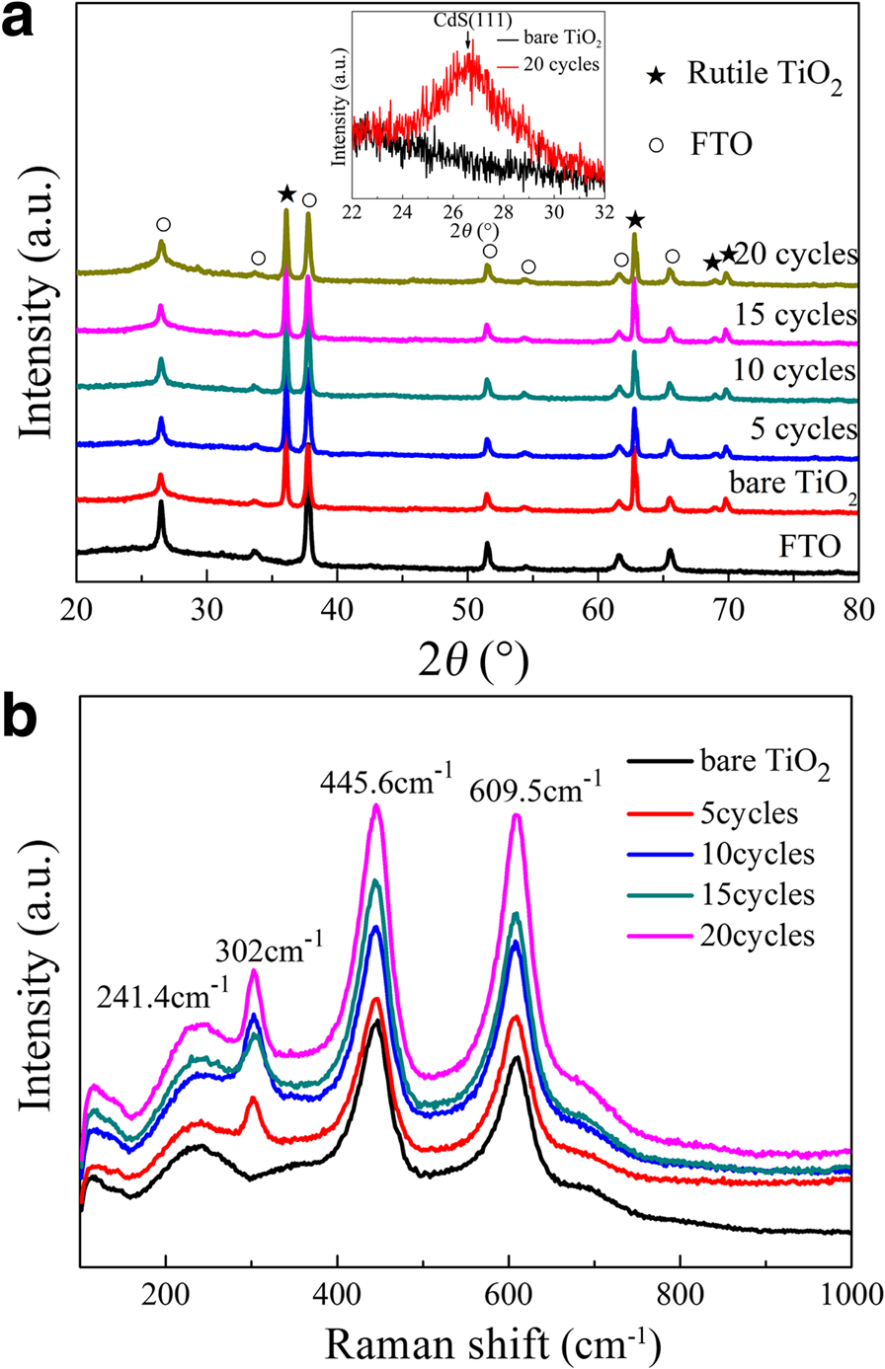
**a** XRD patterns and **b** Raman spectra of TiO_2_ NRAs/CdS

The XRD patterns of TiO_2_ NRAs/CdS are shown in Fig. 1a. The characteristic peaks at 2*θ* = 36.078°, 62.750°, 69.010°, and 69.795° can be indexed to rutile TiO_2_ (PDF No. 21-1276). Other peaks are attributed to the FTO substrate. There is no characteristic peak for CdS after SILAR, and the absence of diffraction peak associated with CdS might be due to the low concentration and the well dispersion of CdS in the nanocomposite. To further confirm the presence of CdS, we measured the bare TiO_2_ NRAs and the TiO_2_ NRAs/CdS (20 cycles) samples with the glancing angle X-ray diffraction (GXRD) method. The GXRD measurement was performed with a scanning step of 0.02° and a dwell time of 0.15 s in the scanning range of 22°–32°. The corresponding GXRD pattern is shown in the inset of Fig. 1a. It can be seen that only the TiO_2_ NRAs/CdS (20 cycles) sample displays one peak at about 26.5°, which is corresponding to CdS (111) (PDF No. 10-0454) and confirms the successful deposition of CdS on TiO_2_ NRAs.

Raman microscopy was conducted to further identify the presence and crystallinity of CdS, and the results are displayed in Fig. 1b. The peak at 117 cm^−1^ is due to plasma emission of the Ar^+^ laser [21]. The three strong Raman peaks located around 241.4, 445.6, and 609.5 cm^−1^ should be assigned to the Raman active modes of rutile TiO_2_ [22], which is consistent with the XRD patterns. The well-resolved band located at ~302 cm^−1^ is from CdS [23], which is in accordance with the first-order scattering of the longitudinal optical phonon mode [24].

**Fig. 2:**
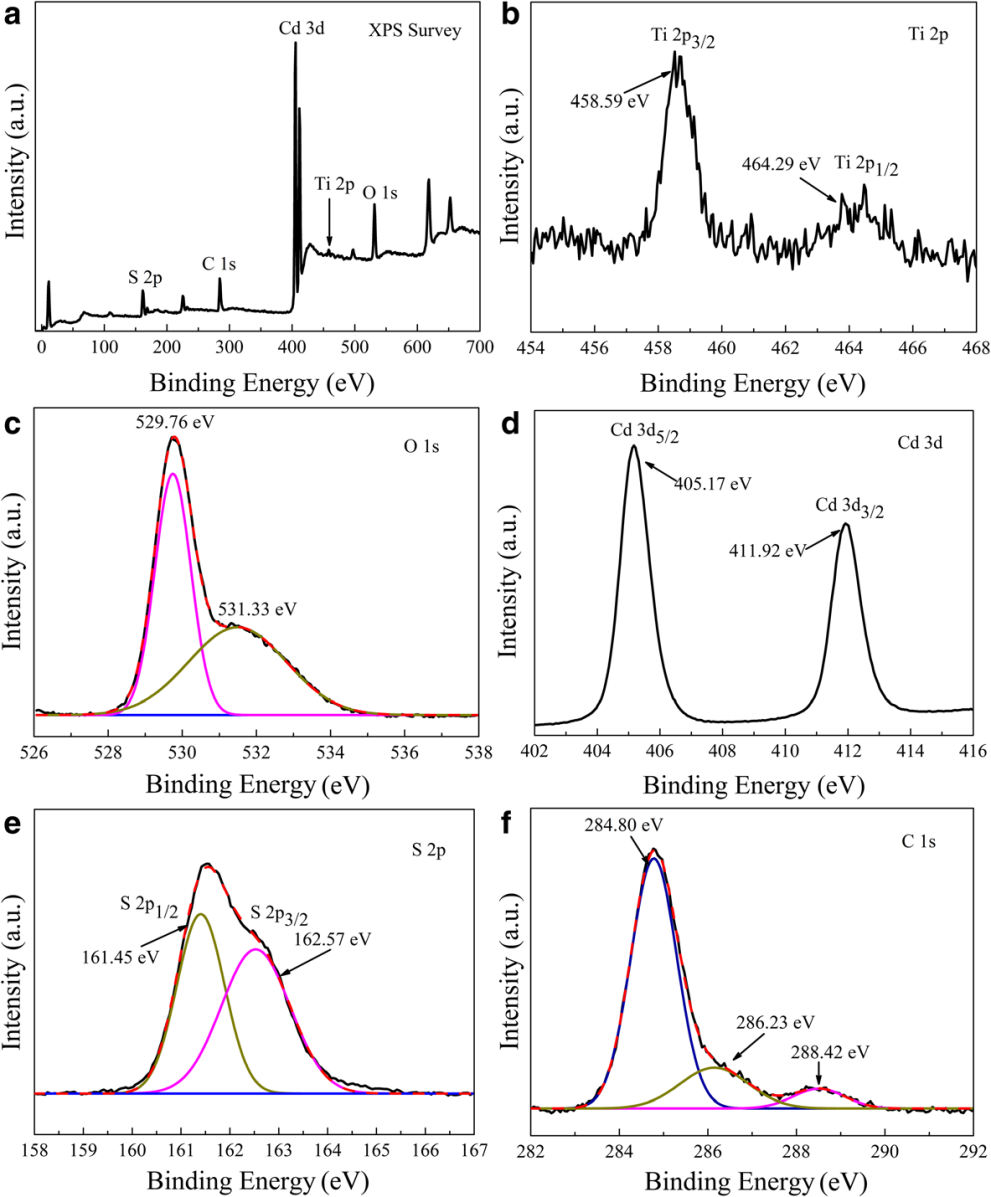
**a** XPS survey spectrum of TiO_2_/CdS (15 cycles) and high-resolution XPS spectra of **b** Ti 2p, **c** O 1s, **d** Cd 3d, **e** S 2p, and **f** C 1s

To further reveal the valence states and surface chemical compositions of the composite, XPS is employed to characterize the TiO_2_ NRAs/CdS (15 cycles) sample. Figure 2a confirms Ti, O, Cd, S, and C are present in the nanocomposite. In Fig. 2b, two peaks for the Ti 2p are observed (464.29 eV for Ti 2p_1/2_ and 458.59 eV for Ti 2p_3/2_). These values are in good agreement with the XPS data known for Ti^4+^ in TiO_2_ [25]. The high-resolution spectrum of O 1s in Fig. 2c shows two components by Gaussian curve fittings. The pronounced peak at 529.76 eV is attributed to the lattice oxygen of TiO_2_, and the other peak at 531.33 eV is attributed to oxygen defect (i.e., Ti–OH) [26]. It is reported that oxygen defect may play an important role in enhancing the photocatalytic activity [27]. Two bands at 405.17 and 411.92 eV are observed in Fig. 2d, which can be ascribed to the Cd 3d_5/2_ and Cd 3d_3/2_ binding energies, respectively. The result is accordance with the previous report of Cd^2+^ values [28]. Moreover, XPS peaks of S 2p located at 161.45 and 162.57 eV should be assigned to the spectra of S 2p_1/2_ and S 2p_3/2_, respectively, indicating that the composite electrode contains S^2+^ of CdS [29]. As to the high-resolution spectrum of O 1s shown in Fig. 2f, the peak at 284.80 eV is from adventitious carbon (C–C/C–H bonds), which is inevitable in XPS measurement [30], while the peaks at 286.23 and 288.42 eV may be due to the formation of carbonate species [31, 32]. From the above analysis, one can clearly see that CdS is successfully deposited on the TiO_2_ NRAs.

**Fig. 3:**
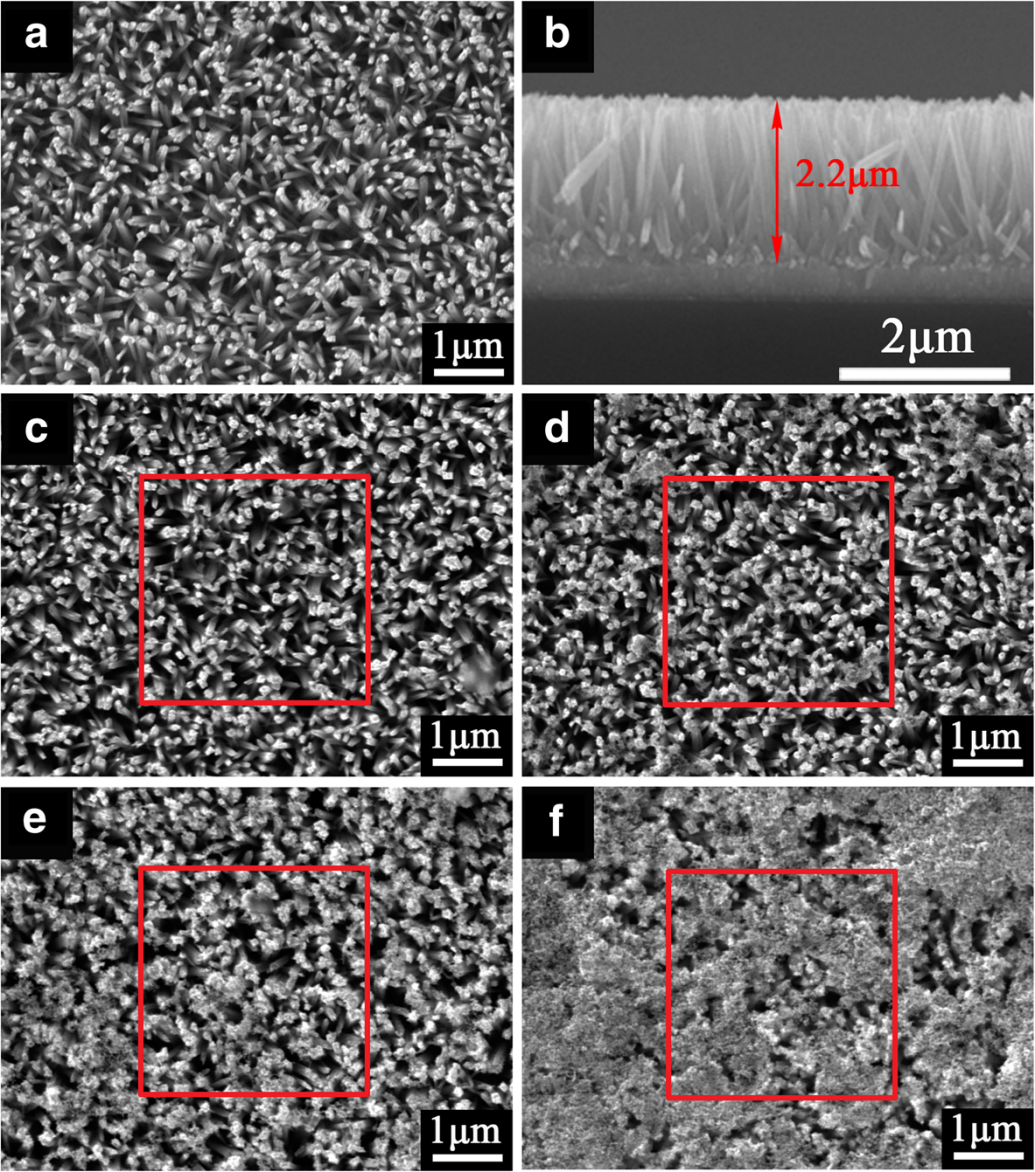
SEM images of TiO_2_ NRAs/CdS: **a** 0, **c** 5, **d** 10, **e** 15, and **f** 20 SILAR cycles. **b** The corresponding cross section image of **a**

The top-view SEM image of the bare TiO_2_ NRAs is shown in Fig. 3a. It can be seen that the nanorods are uniform with a rectangular cross section and the nanorod diameter is around 60~120 nm. The corresponding cross section image (in Fig. 3b) shows that vertically or slantingly aligned nanorod arrays are uniformly grown in high density on the FTO substrate and the typical nanorod length was about 2.2 μm. From Fig. 3b–f, it can be seen that the amount of CdS accumulated gradually with increase in SILAR cycles. Especially when TiO_2_ NRAs were decorated with CdS by 20 cycles (in Fig. 3f), the entire surface of the TiO_2_ NRAs was almost covered by a film consisting of larger CdS crystallites. EDS analysis was also carried out for areas marked by the red rectangles in Fig. 3. The results are shown in Additional file 1: Figure S1. It is observed that the Ti/Cd ratio was from ∼23.94 to ∼3.31 when the SILAR cycles increased from 5 to 20 cycles, which indicates that more CdS NPs were deposited on TiO_2_ NRAs with a higher number of SILAR cycles.

**Fig. 4:**
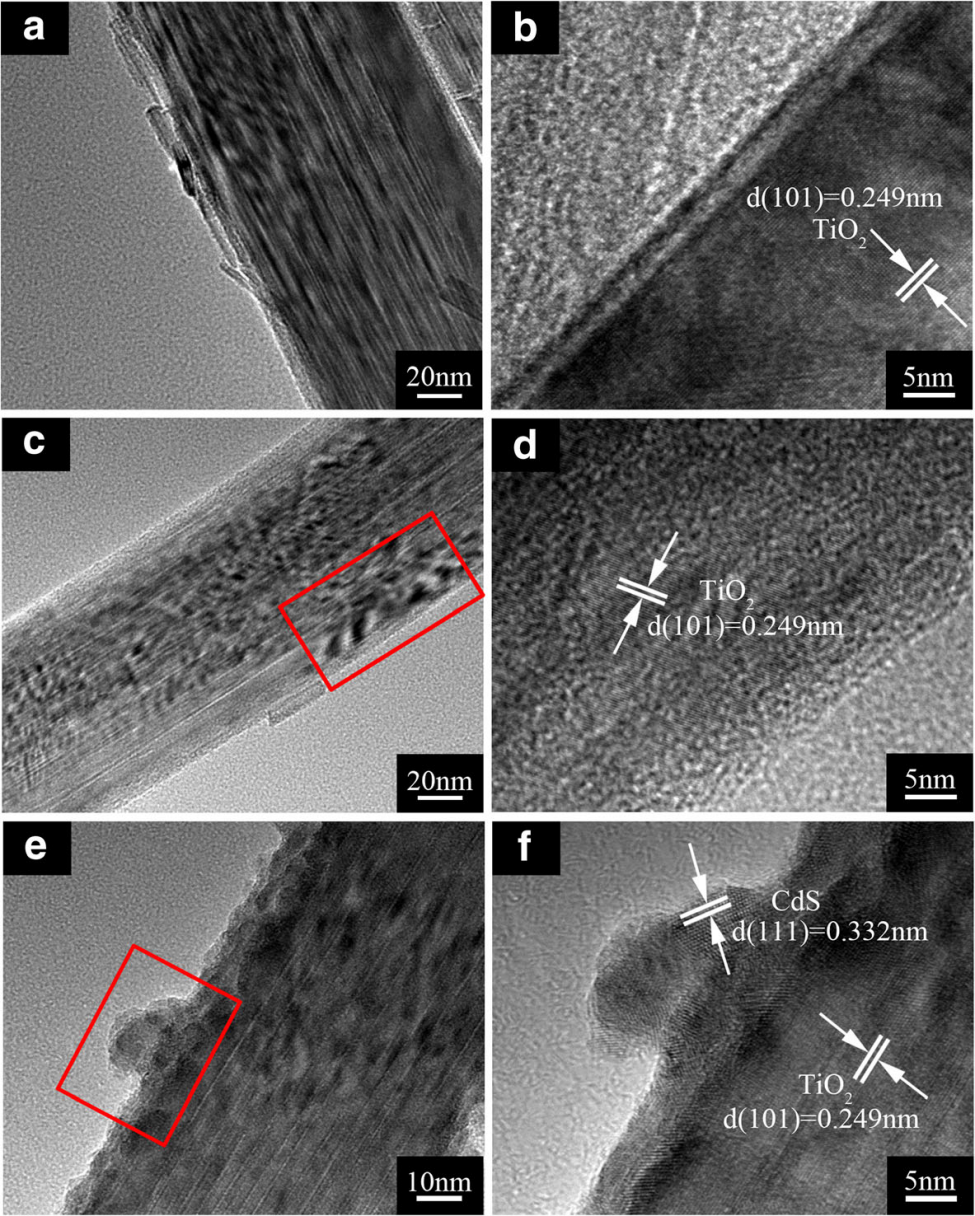
TEM images. **a**, **b** The bare TiO_2_ NRAs. **c**, **d** TiO_2_ NRAs/CdS (5 cycles). **e**, **f** TiO_2_ NRAs/CdS (15 cycles)

The morphology of TiO_2_ NRAs/CdS was further investigated by TEM. From Fig. 4a, c, e, it can be seen that the diameter of the nanorod in all of the samples is consistent with the result in SEM images. The corresponding high-resolution TEM images all show the lattice fringes of rutile TiO_2_. From the TEM images of the TiO_2_ NRAs/CdS (5 cycles) in Fig. 4c, d, no obviously recognizable CdS NPs on the surface of the nanorods can be found due to the low content of CdS after only 5 cycles’ deposition. However, Additional file 1: Figure S1 (a) displays the existence of CdS in the TiO_2_ NRAs/CdS (5 cycles) sample. When the SILAR deposition increased to 15 cycles shown in Fig. 4e, f, it can be seen that the smooth surface of the bare TiO_2_ NRAs become rough after the deposition of CdS. A thin layer made of CdS particles covered the whole nanorod as shown in Additional file 2: Figure S2. The high-resolution TEM image in Fig. 4f gives a lattice fringe of about 0.332 nm, corresponding to the *d* (111) space of CdS.

**Fig. 5:**
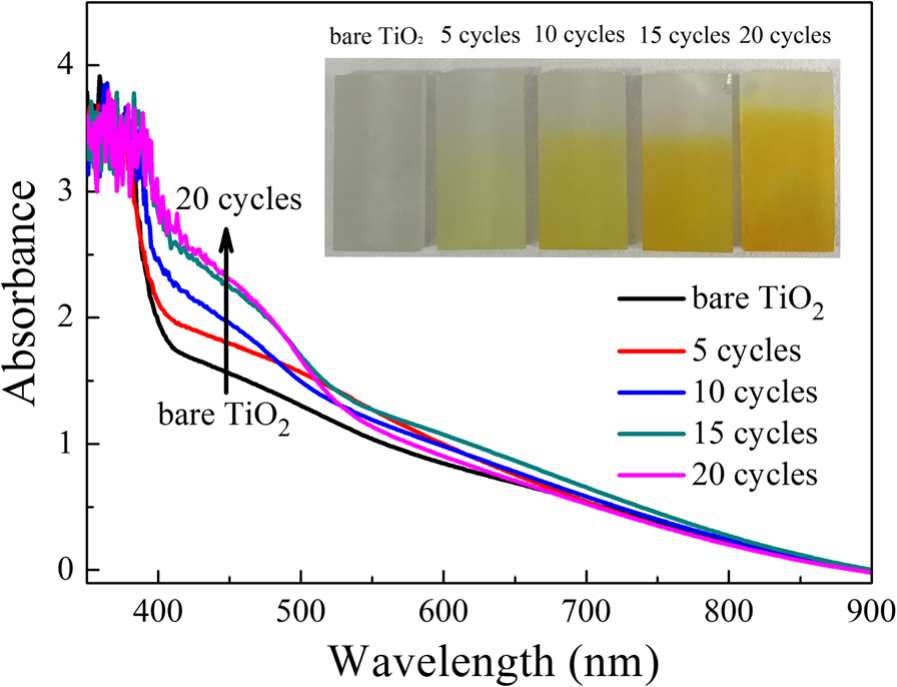
Absorption spectra of TiO_2_ NRAs/CdS. *Inset* is the optical photograph of TiO_2_ NRAs/CdS

The optical absorption property of TiO_2_ NRAs decorated with CdS NPs is shown in Fig. 5. The inset photograph is the image of the TiO_2_ NRAs/CdS NPs, showing clearly the color change with different SILAR cycles. With the deposition of CdS NPs, light absorption of TiO_2_ NRAs was strengthened from 400 to 500 nm. The more CdS are deposited, the stronger the visible light absorption capacity is. It is reported that any red shift in optical response of TiO_2_ toward the longer wavelength region gives the possibility of higher photocatalytic activity [33]. Tiny absorption of the as-prepared TiO_2_ sample in the visible light range was found. This abnormal phenomenon can be attributed to the scattering of light caused by the nanorod arrays as well as the absorption by the FTO itself [29, 34].

**Fig. 6:**
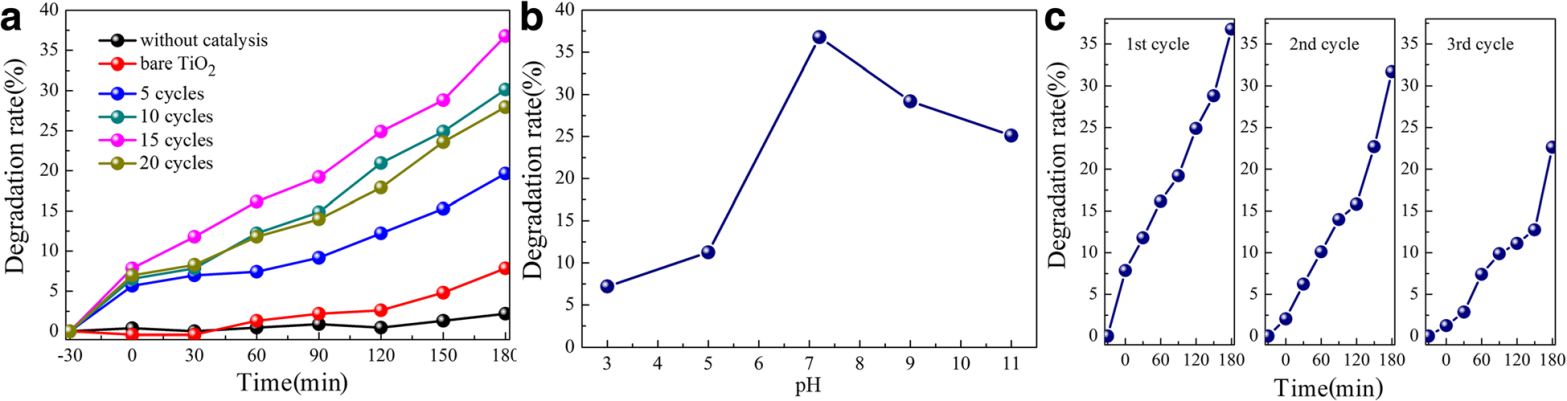
**a** Degradation rate of UDMH by TiO_2_ NRAs/CdS with different SILAR cycles. **b** Influence of different pH on the degradation rate of UDMH. **c** Cyclic photodegradation of UDMH with TiO_2_ NRAs/CdS (15 cycles)

Photodegradation of UDMH was carried out under visible light irradiation (*λ* ≥ 420 nm) using the CdS-decorated TiO_2_ NRAs as a photocatalyst. In Fig. 6a, with the extension of the visible light irradiation time, one can see that the degradation rates of UDMH by different photocatalysts increased. Without the addition of any catalysts under visible light irradiation for 180 min, the degradation rate of UDMH was only 2.18%, which indicated that the degradation ability by simple visible light was very low, while the bare TiO_2_ NRAs could achieve 7.86% under the same condition. Enhancement could be observed by using the CdS-decorated TiO_2_ NRAs as the photocatalyst. For example, the degradation rate was 19.56% by TiO_2_ NRAs/CdS (5 cycles), and it could reach 36.77% if using TiO_2_ NRAs/CdS (15 cycles). However, continually increasing the SILAR cycles to 20, the degradation rate decreased (i.e., it was down to 27.95%) instead of getting higher. It can be seen that the degradation rate of UDMH first increased with the increase of the CdS deposition cycles and then decreased when the CdS deposition cycles continually increased. Thus, we may deduce that when a proper amount of CdS NPs are decorated, more visible light could be absorbed to produce more excited carriers and the carriers could be separated more efficiently, which lead to higher degradation rates. However, excess deposition of CdS NPs in the TiO_2_ NRAs/CdS (20 cycles) sample causes a longer transport path for the photogenerated electron–hole pairs [15, 35], and it is a potential barrier for charge carrier transfer. This is not beneficial for the effective separation of carriers, therefore leading to the decrease of the photodegradation rate. A proper amount of CdS decoration is the key factor that decides the photodegradation efficiency.

The influence of pH on the degradation rate of UDMH was also studied, and pH of the simulated UDMH solution was adjusted to 3, 5, 9, and 11 by NaOH and H_2_SO_4_. In this experiment, TiO_2_ NRAs decorated with CdS NPs by 15 SILAR cycles were chosen as the catalyst. It was discovered that the best degradation rate of UDMH could be achieved in the neutral solution (pH is c.a. 7.2). The photocatalytic degradation rate of UDMH under alkaline circumstance is better than that under acid circumstance.

Durability is another important point of CdS-related photocatalyst because CdS may cause photocorrosion under irradiation. The visible photocatalytic durability of the TiO_2_ NRAs/CdS (15 cycles) sample was investigated and displayed in Fig. 6c. The photodegradation ratio of UDMH after each 180-min irradiation for 3 cycles was about 36.77, 31.69, and 22.63%, respectively. Photocorrosion effect led to more than 35% decrease of the degradation rate after three runs. It means that part of Cd^2+^ was left in the aqueous solution which could result in the second pollution, the most pressing problem about CdS-related photoactivity. However, for the excellent photoconversion of CdS, it still attracts intensive studies by lots of researchers. In our previous experiment [36], NiFe_2_O_4_-modified TiO_2_ NRAs were used in the photodegradation of UDMH wastewater under the same condition; however, the degrading rate (c.a. 22.06%) was relatively low compared with that of CdS-modified TiO_2_ NRAs. In this case, we would be committed to improve the durability of TiO_2_ NRAs/CdS in the future.

A proposed model for the photodegradation activity can be illustrated as follows. When TiO_2_ NRAs/CdS is irradiated by visible light (*λ* ≥ 420 nm), CdS could be effectively excited to produce electron and hole pairs. As the conduction band (CB) of TiO_2_ is more positive than that of CdS, the excited electrons immigrate from the CB of CdS to the CB of TiO_2_. Thus, the photoinduced charge carriers can be effectively separated, and the lifetime is prolonged. The accumulated electrons (*e* ^−^) in the CB of TiO_2_ could react with dissolved oxygen molecules to form superoxide radical anions (·O_2_−) [37], which could be further reduced to highly reactive hydroxyl radicals (·OH) [38]. The positive holes in the valence band (VB) of CdS can also be trapped by OH^−^ to produce ·OH species [39]. These strong oxidizing free radicals then react with UDMH. Under acid circumstance, abundant H^+^ existing in the resolution may hinder process (3), while under alkaline circumstance, OH^−^ existing in the resolution may be in favor of process (4); this may account for the effect of the pH on the degrading rate.

1 $$ \mathrm{C}\mathrm{d}\mathrm{S} + \mathrm{h}\upnu \left(\mathrm{visible}\right)\ \to\ \mathrm{C}\mathrm{d}\mathrm{S}\left({e_{\mathrm{cb}}}^{-} + {h_{\mathrm{vb}}}^{+}\right) $$

2 $$ \mathrm{C}\mathrm{d}\mathrm{S}\left({e_{\mathrm{cb}}}^{-} + {h_{\mathrm{vb}}}^{+}\right) + {\mathrm{TiO}}_2\ \to\ \mathrm{C}\mathrm{d}\mathrm{S}\left({h_{\mathrm{vb}}}^{+}\right) + {\mathrm{TiO}}_2\left({e_{\mathrm{cb}}}^{-}\right) $$

3 $$ {h_{\mathrm{vb}}}^{+}+{\mathrm{H}}_2\mathrm{O}\ \to\ \cdotp \mathrm{O}\mathrm{H} + {\mathrm{H}}^{+} $$

4 $$ {h_{\mathrm{vb}}}^{+}+{\mathrm{HO}}^{-}\ \to\ \cdotp \mathrm{O}\mathrm{H} $$

5 $$ {\mathrm{TiO}}_2\left({e_{\mathrm{cb}}}^{-}\right)+{\mathrm{O}}_2\ \to\ {\mathrm{TiO}}_2+\cdot {{\mathrm{O}}_2}^{-} $$

6 $$ \cdotp\ \mathrm{O}\mathrm{H} + \mathrm{UDMH}\ \to\ \mathrm{degradation}\ \mathrm{products} $$

7 $$ \cdot {{\mathrm{O}}_2}^{-}+\mathrm{UDMH}\ \to\ {{\mathrm{degradation}\ \mathrm{products}\ \mathrm{or}\kern0.8em \cdot \mathrm{O}}_2}^{-}+{\mathrm{H}}^{+}\ \to\ {\mathrm{H}\mathrm{O}}_2 \cdot \to\ \to \cdot \mathrm{O}\mathrm{H} $$

To better understand the photocatalytic performance of the TiO_2_ NRAs/CdS, photocurrent intensity versus potential (*I*–*V*) and PL measurements were carried out.

**Fig. 7:**
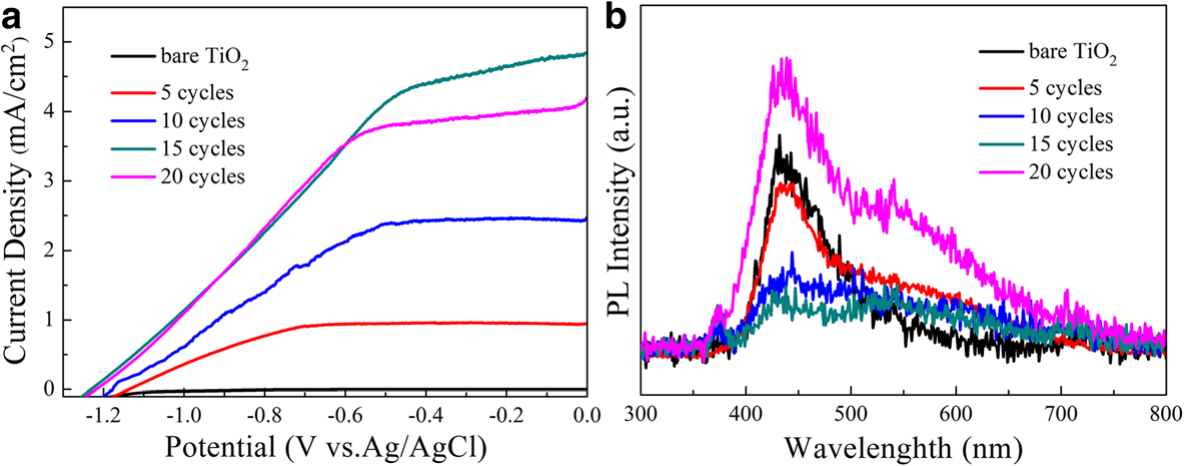
**a** Photocurrent intensity versus potential characteristics of the TiO_2_ NRAs/CdS NPs. **b** PL spectra of TiO_2_/NRAs CdS NPs excited by 350 nm

Figure 7a shows the *I*–*V* curves measured for TiO_2_ NRAs/CdS. Under visible light irradiation, the bare TiO_2_ NRAs electrode showed little photocurrent density. After the deposition of CdS, the photocurrent density of the samples increased remarkably. A higher photocurrent density indicates a higher efficiency in the separation of electrons and holes [40], thus suggesting a better photocatalytic performance. In Fig. 7a, the photocurrent density first increased with the increase of the CdS NPs deposited from 5 to 15 cycles. However, when the deposition of CdS reached to 20 cycles, the photocurrent density was significantly decreased rather than continuing to increase. Though more CdS NPs brought the increased harvesting of photons, it did not lead to the continual increase of the photocurrent density. Two points may account for this phenomenon. Firstly, excess CdS deposition made the CdS crystallites larger, which increased the transfer path for the photogenerated carriers [15, 35] and thus hindered the fast transport of the carriers. Secondly, when the SILAR deposition increased to 20 cycles, the abrupt increase of CdS nanoparticles would create more defects, which could act as recombination centers [29]. The two points both result in the ineffective separation of the carriers. In addition, Fig. 7a also shows that the open circuit potential (*V*_oc_) for TiO_2_ NRAs become more negative after decorated with CdS (−0.26 V for the bare TiO_2_ NRAs, −1.13, −1.17, −1.23, and −1.21 V for TiO_2_ NRAs decorated by 5, 10, 15, and 20 cycles, respectively). It is reported that more negative *V*_oc_ means better charge carrier separation [41, 42], thus leading to better photocatalytic capacity.

The PL technique is an effective way to explore the separation of the charge carriers [29]. Figure 7b demonstrates the PL spectra of the CdS-decorated TiO_2_ NRAs, which are excited at a wavelength of 350 nm. The peak in the PL spectra originates from the recombination of the photogenerated electron–hole pairs [29, 43]. The higher the PL intensity is, the higher the recombination rate of the carriers is [43, 44]. It is clear to see that a broad emission peak centered at around 450 nm was observed for all the samples. With the SILAR cycles increased to 15 cycles, the intensity of the emission peak is quenched drastically. This indicates that the introduction of CdS brings more effective separation of the photoinduced electron–hole carrier pairs, the prolonged lifetime of the carriers, and thereby the less recombination rate of the photogenerated electron and holes in the TiO_2_/CdS nanocomposite [45]. However, continually increasing the SILAR to 20 cycles, there would be an abrupt increase of CdS nanoparticles to form defects. These defects could act as recombination centers for the photoinduced carriers [29, 46], thus initiating the rapid photoelectron–hole recombination within CdS [47] and therefore causing a stronger PL intensity.

From the *I*–*V* curves and PL spectra, it can be seen that a proper amount of CdS decoration makes more effective charge carrier separation, which will then play an important role in the following photocatalytic degrading activity.

## Conclusions

The CdS NP-decorated TiO_2_ NRAs were synthesized and applied for the photodegradation of UDMH under visible light irradiation. Compared with the bare TiO_2_ NRAs, TiO_2_ NRAs/CdS heterojunction exhibited enhanced photocatalytic capacity toward UDMH. By adjusting the cycles of SILAR, TiO_2_ NRAs decorated by 15 cycles of CdS got the best degradation efficiency of UDMH. Besides, it seems that alkaline circumstance is more beneficial for the photocatalytic degradation of UDMH than acid circumstance. When the pH of the simulated UDMH wastewater was about 7.2, the degradation rate of UDMH was highest. Through *I*–*V* and PL characterization, the proposed photocatalytic mechanism was further confirmed. The synergetic effect between CdS and TiO_2_ leads to high electron injection efficiency and fast electron transfer; thus, the photocatalytic capacity of TiO_2_ NRAs/CdS can be enhanced significantly. This research proved that photocatalysis may be a possible way to deal with the toxic UDMH wastewater with low energy consumption and easy recycle of the catalyst.

## Additional files

Additional file 1: Figure S1. EDS images of TiO_2_ NRAs/CdS. (a) 5 cycles, (b) 10 cycles, (c) 15 cycles, and (d) 20 cycles. (448 KB)
Additional file 2: Figure S2. TEM image of TiO_2_ NRAs/CdS (15 cycles). (964 KB)

## Abbreviations

CB: Conduction band
DI: Deionized water
EDS: Energy-dispersive X-ray spectroscopy
FTO: Fluorine-doped tin oxide
GXRD: Glancing angle X-ray diffraction
*I*–*V*: Photocurrent intensity versus potential
NPs: Nanoparticles
PL: Photoluminescence
SILAR: Successive ion layer adsorption and reaction
TEM: Transmission electron microscopy
TiO_2_ NRAs: TiO_2_ nanorod arrays
UDMH: Unsymmetrical dimethylhydrazine
VB: Valence band
*V*_oc_: Open circuit potential
XPS: X-ray photoelectron spectroscopy
XRD: X-ray diffraction
