# Supplementary figures and images for: Photodegradation of Unsymmetrical Dimethylhydrazine by TiO2 Nanorod Arrays Decorated with CdS Nanoparticles Under Visible Light
Source: Nanoscale Res Lett. 2016 Nov 10;11:496. doi: 10.1186/s11671-016-1718-9 (PMC5104699; doi:10.1186/s11671-016-1718-9)

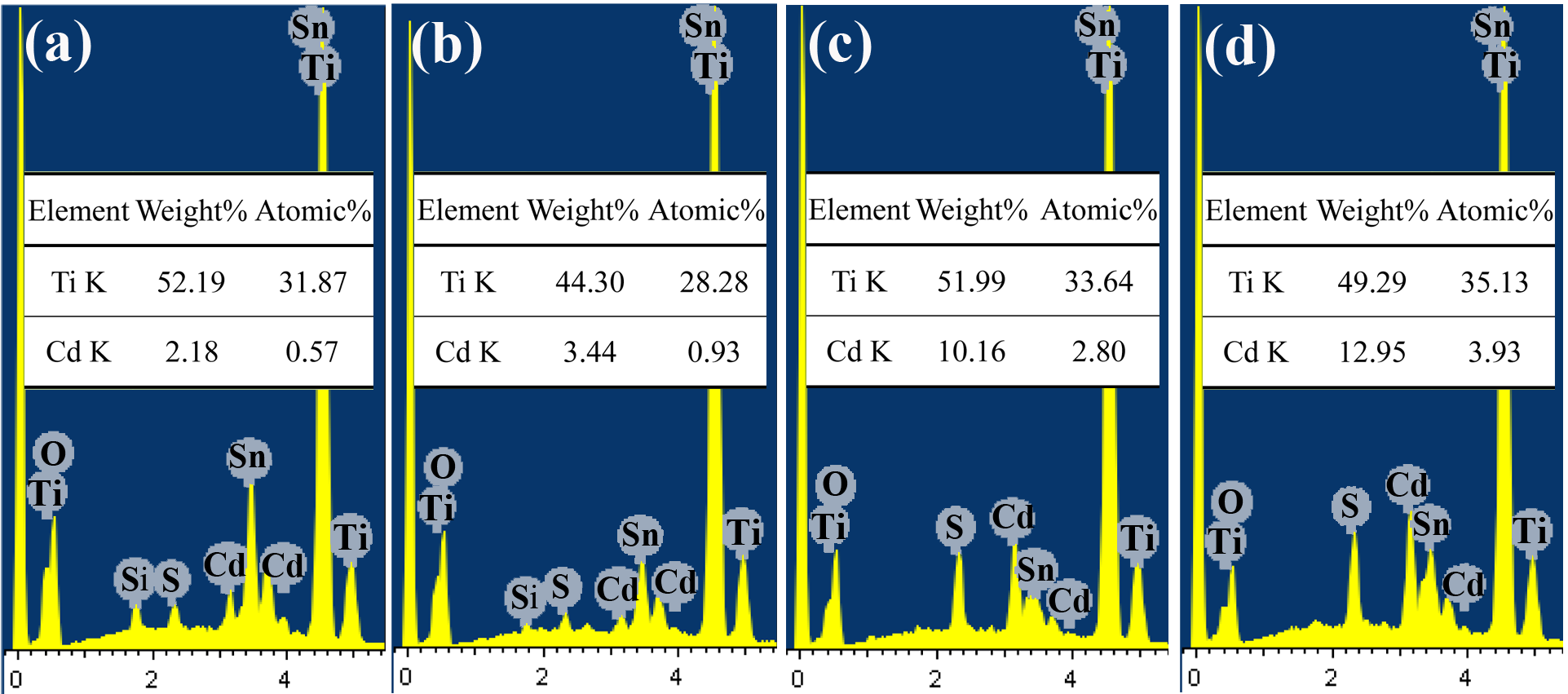

Supplement: Additional file 1: Figure S1. — EDS images of TiO2 NRAs/CdS. (a) 5 cycles, (b) 10 cycles, (c) 15 cycles, and (d) 20 cycles. (448 KB) [file 11671_2016_1718_MOESM1_ESM.tif]

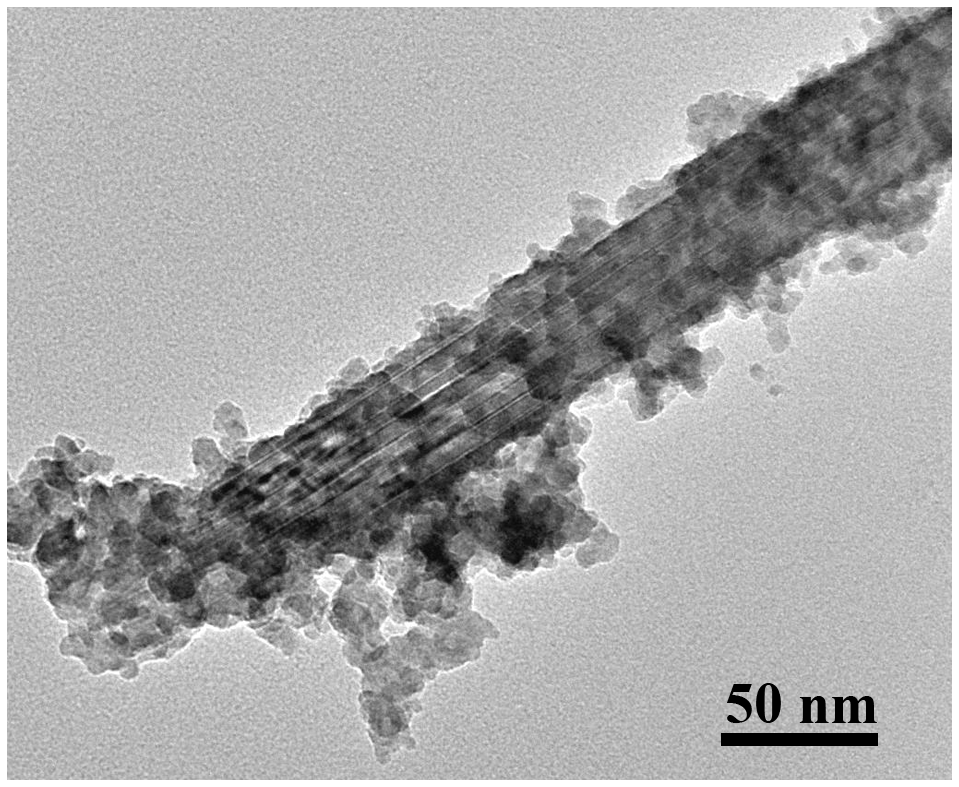

Supplement: Additional file 2: Figure S2. — TEM image of TiO2 NRAs/CdS (15 cycles). (964 KB) [file 11671_2016_1718_MOESM2_ESM.tif]
